# The sequencing and characterization of chloroplast genome of *Geranium sibiricum* Linne

**DOI:** 10.1080/23802359.2021.1947912

**Published:** 2021-07-06

**Authors:** Sun Huizhen, Xinyuan Xue

**Affiliations:** Center for Ecological Research, Northeast Forestry University, Harbin, P.R. China

**Keywords:** *Geranium sibiricum*, chloroplast genome, phylogenetic analysis

## Abstract

The complete chloroplast genome of *Geranium sibiricum* Linne. was sequenced, assembled and annotated. It is a circular form of 150,656 bp in length, which was separated into four distinct regions, a large single copy (LSC) of 73,862 bp, a small single copy region (SSC) of 52,666 bp, two inverted repeats (IR) of 12,064bp. A total of 124 genes were predicted, of which, 87 encode proteins, 4 rRNA, 33 tRNA. The evolutionary history was inferred using Maximum Likelihood method, and the result indicates that *G. sibiricum* was grouped within Geraniaceae, and comprised a clade with *Geranium palmatum* under 100% Bootstrap value.

*Geranium sibiricum* L., belonging to Geraniaceae, is a widely distributed herb in northern China, Korea, Japan, and some European countries (Shim et al. 2009; Wu et al. 2010). This herb is used as food, or condiment for cooking meat in some countries (Wu et al.^2010^). For containing bioactive constituents, such as geraniin, corilagin, and gallic acid, it is also used as medicines for healing bacteria, intestinal inflammation, dermatitis, diarrhea, cancer and for hair growth-promoting (Wang et al. 2015; Shim and Lim 2008, 2009; Shim et al. 2009; Wu et al.^2010^; Boisvert et al. 2017). In previous studies, although many literatures on chemical composition and their functions have been documented, there were hardly any its genetic researches. In this study, we report the complete chloroplast (cp) genome of *G. sibiricum*.

Samples were collected from Qilian mountains (36°35′12″N, 101°49′11″E) in Qinghai province. Voucher specimen (LQ2020060506) was deposited in the Herbarium, Center for Ecological Research, Northeast Forestry University. A sample’s total genomic DNA was extracted from about 100 mg fresh leaves using a modified CTAB method (Murray and Thompson 1980). An average length of 350 bp Paired-end Libraries with NexteraXT DNA Library Preparation Kit (Illumina, San Diego, CA) was constructed and sequenced on Illumina Novaseq 6000 platform (Shenzhen Huitong biotechnology Co. Ltd). Raw sequence reads were filtered using NGS QC Toolkit (Patel and Jain 2012) and 5.86 Gb clean data was de novo assembled by SPAdes v.3.9.0 soft-ware (Bankevich et al. 2012) with the chloroplast genome of *Geranium incanum* Burm. f. (Accession number: KT760575.1) as reference. The assembled complete cp genome was annotated via PGA (Qu et al. 2019).

The complete cp genome of *G. sibiricum* (GenBank accession no. MW266075.1) is quadripartite form of 150,656bp in length, and composed of a large single copy region (LSC, 73,862 bp), a small single copy region (SSC, 52,666 bp), two inverted repeats (IR, 12,064 bp). GC content of the genome is 38%. A total of 124 genes were predicted on this cp genome, of which, 87 encode proteins, 4 rRNA, 33 tRNA.

Phylogenetic analysis was performed based on complete cp genomes of *G. sibiricum* and other 18 related species reported in Geraniaceae, three species in Malvaceae as out-group. The sequences were aligned using HomBlocks (Bi et al. 2018). The evolutionary history was inferred with Maximum Likelihood (ML) method using MEGA X under GTR + G + I model, and partial deletion of gaps/missing data (Kumar et al. 2018). Bootstrap (BS) values were calculated from 1000 replicate analysis (Figure 1). As expected, *G. sibiricum* was grouped within Geraniaceae, and comprised a clade with *Geranium palmatum* under 100% BS value. The complete cp genome of *G. sibiricum* will be helpful for further studies on molecular biology, population genetics, taxonomy or resources protection.

**Figure 1.**
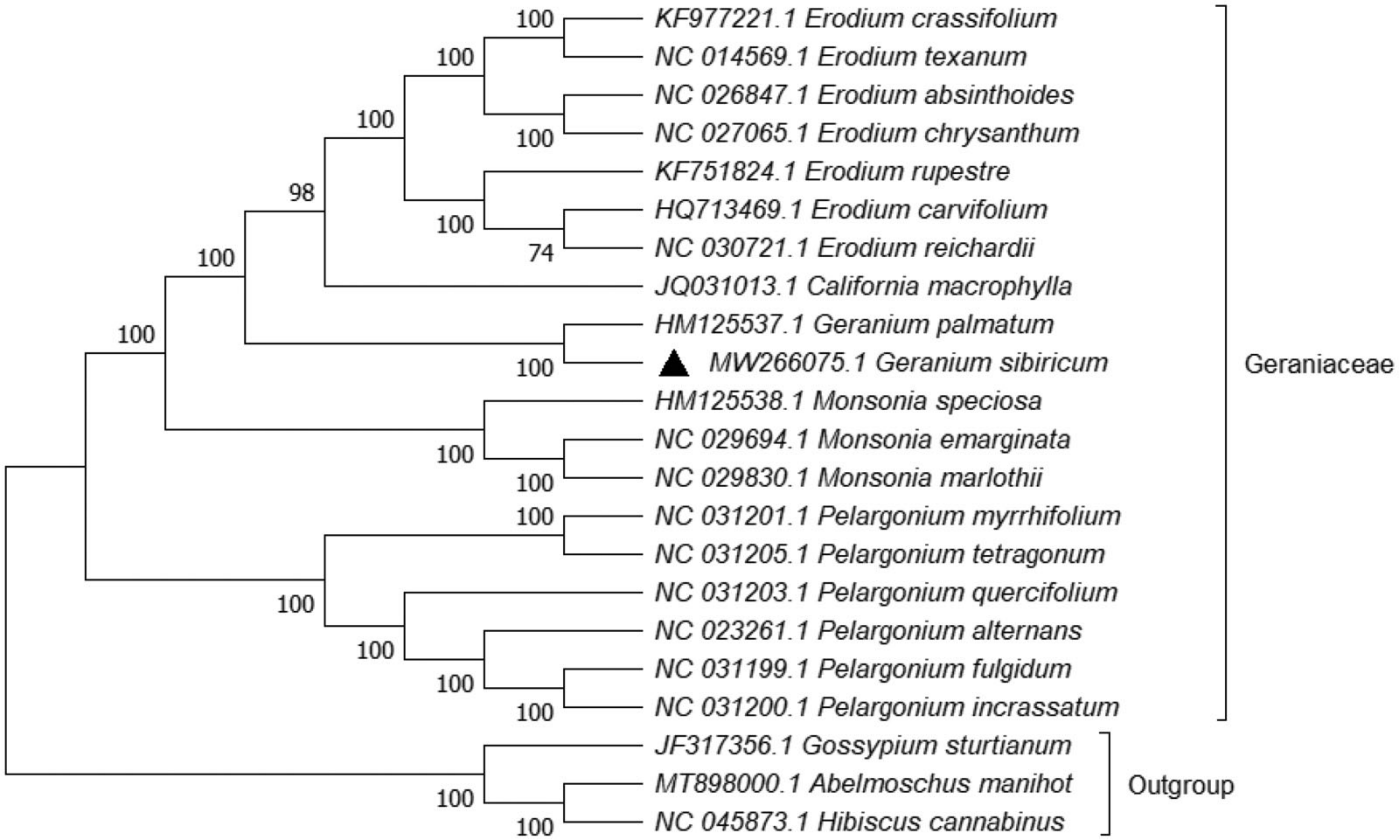
ML phylogenetic tree based on 22 species chloroplast genomes was constructed using MEGA X. Numbers on each node are bootstrap from 1000 replicates.

## Data Availability

The genome sequence data that support the findings of this study are openly available in GenBank of NCBI at (https://www.ncbi.nlm.nih.gov/nuccore/MW266075.1) under the accession no. MW266075.1. The associated BioProject, SRA, and Bio-Sample numbers are PRJNA705079, SRR13789010, and SAMN18062704, respectively.
